# Mammographic density and breast tissue expression of inflammatory markers, growth factors, and vimentin

**DOI:** 10.1186/s12885-018-5088-9

**Published:** 2018-11-29

**Authors:** Gertraud Maskarinec, Dan Ju, Jaimie Fong, David Horio, Owen Chan, Lenora W. M. Loo, Brenda Y. Hernandez

**Affiliations:** University of Hawaii Cancer Center, 701 Ilalo Street, Honolulu, HI 96813 USA

**Keywords:** Breast tissue, Tissue microarray, Immunohistochemistry, Mammographic density

## Abstract

**Background:**

Mammographic density is a known risk factor for breast cancer, but the underlying pathologic characteristics are not well understood. The current analysis investigated the expression of several markers of interest, e.g., inflammation and growth, with mammographic density (MD) in normal and malignant breast tissue specimens from 279 women of the Multiethnic Cohort (MEC).

**Methods:**

Breast cancer cases, recruited from a nested case-control study within the MEC, provided mammograms for density evaluation. Protein expression (COX-2, TNF-α, TGF-β, IGF-1R, IGFBP-2, and vimentin) was assessed by immunohistochemical detection. Linear regression was applied to evaluate the relation between marker expression and percent density and to compute adjusted means with 95% confidence intervals (CI) by marker status while adjusting for confounders.

**Results:**

Due to missing cores and tissue, normal tissue could only be evaluated for COX-2 and vimentin. No significant associations with mammographic density were detected for all markers analyzed. For inflammatory markers (TNF-α, COX-2, and TGF-β) in tumor tissue, MD were non-significantly higher with stronger expression but the differences were very small. For example, the mean MD values for no, weak, and strong TNF-α expression were 35% (95% CI 24–47%), 39% (95% CI 29–48%), and 38% (95% CI 27–50%). In a *posthoc* analysis among postmenopausal women only, the difference across categories of TNF-α expression increased to 25% (95% CI 12–39%), 35% (95% CI 23–48%), and 35% (95% CI 20–49%).

**Conclusions:**

The current analysis offers little support for an involvement of immunohistochemical markers representing inflammatory and growth factor pathways as predictors of breast density.

**Electronic supplementary material:**

The online version of this article (10.1186/s12885-018-5088-9) contains supplementary material, which is available to authorized users.

## Background

Although mammographic density (MD) is known as a strong predictor for breast cancer development [1], the underlying histopathology of MD and its role in oncogenesis remain unclear [2]. The dense areas of the breast, composed of epithelial and stromal tissue, are hypothesized to represent cumulative exposure to hormones and growth factors [3, 4]. Several markers of inflammation have been related to breast cancer risk and outcome. Cyclooxygenase-2 (COX-2) expression in breast tumor tissue has been associated with poor breast cancer prognosis [5] as well as high serum TNF-α and TGF-β levels [6]. The leukocyte common antigen CD45 is a transmembrane protein commonly found on immune cells and has been shown to be a marker for poor prognosis in small cell lung carcinoma [7]. Other markers involved in cellular proliferation, such as members of the insulin-like growth factor (IGF) protein family, and in structural and physiological processes of the breast, including vimentin and STAT3, have been implicated in the pathogenesis of cancer. Elevated circulating levels of IGF-1/IGFBP-3 and IGF-1R in triple-negative breast cancer are associated with worse survival [8, 9], while IGFBP-2 has been associated with better survival [10]. STAT3, while necessary for mammary gland involution, is found in most breast tumors, especially triple negative cancers [11], and nuclear localization of STAT3 expression appears to be a favorable prognostic marker in patients with invasive breast cancer [12, 13]. Vimentin is found in all stromal cells and helps to preserve cellular integrity; increased DNA methylation of its gene predicted poorer overall survival in breast cancer patients [14]. Breast tissue with higher mammographic density was found to have a significantly greater proportion of stroma and collagen in addition to epithelium and more vimentin/CD45-positive immune cells [15]. Several of these markers have been investigated in the context of MD but, to our knowledge, not IGFBP-2 and STAT3. Based on the hypothesis that markers promoting inflammation and proliferation are positively associated with higher MD, we investigated the relation of MD as assessed in mammographic images with the expression of several markers in normal and malignant breast tissue of women with breast cancer.

## Materials and methods

### Study population

The current pathologic investigation is based on Hawaii participants of the Multiethnic Cohort (MEC) who took part in a nested case-control (NCC) study of MD and breast cancer risk. The MEC was established in 1993–1996 by mailing a self-administered, 26-page questionnaire asking about demographic, anthropometric, and medical factors to men and women ages 45–75 years residing in Hawaii and California [16]. The MEC is linked annually to the statewide Hawaii Tumor Registry (HTR) to identify incident cancer cases. Of the 118,441 female MC participants, the NCC recruited 607 women with breast cancer and 667 controls in Hawaii [17] and collected updated information on hormone therapy (HT), menopausal status, and mammograms. Invitations for the pathology study were mailed to 430 of the 607 cases in the NCC study with tumor blocks at participating hospitals [18, 19], but 56 of the 335 women who agreed to participate did not have sufficient tissue, leaving 279 women for tissue sampling. The Institutional Review Board at the University of Hawaii approved of all study protocols; all women signed informed consent to be part of the NCC and the pathology investigation.

### MD assessment

Mammographic films of study participants from clinics throughout the State of Hawaii were retrieved and digitized using a Kodak LS 85 Film Digitizer (Kodak, Rochester, NY) with a pixel size of 260 μm. For the pathology study, results of craniocaudal views closest to, but before, the date of diagnosis were selected (mean time 3.8 ± 2.8 years before diagnosis). No information on screening vs. diagnostic mammography was available. Using the Cumulus software developed at the University of Toronto, Canada, the scanned images for both breasts were assessed for densities by one reader (G.M.) who was blinded to the case status and time sequence of mammograms [20]. Percent MD was computed as dense area divided by the total breast area. The interclass correlation coefficients derived from duplicate readings (410 out of 5786 images in the original study [17]) were 0.96 (95% CI: 0.95, 0.97) for the size of the dense area and 0.974 for percent density (95% CI: 0.968, 0.978) [20].

### Breast tissue specimens

Pathologic blocks for participants with in situ and invasive breast cancer were retrieved through the tissue repository of the HTR. Information on molecular subtypes was not available. Breast tumor tissue microarrays (TMA) were prepared according to standard procedures [18, 19]. A pathologist identified appropriate tissue blocks from a given patient and prepared a single hematoxylin and eosin (H&E) slide on which representative areas of malignant and normal tissue were marked. The H&E slide was aligned with the corresponding “donor” tissue block and a 0.6 mm cylindrical tissue specimen was taken from the selected area and transferred to a “recipient” paraffin block using a tissue-arraying instrument (Beecher Instruments, Sun Prairie, WI). If available, 4 malignant and 4 normal cores per woman as well as quality controls were placed into 1 of 6 paraffin blocks (maximum 60 sets per block). Out of the 2232 possible specimens, 459 (21%) cores could not be placed due to insufficient tissue in some blocks; this percentage was higher for normal (29%) than malignant (12%) cores. Overall, 1773 (79%) tissue samples with a mean of 6.4 cores per woman were successfully placed. The number of cores that could be evaluated for each marker varied due to tissue composition and type of staining.

### Immunohistochemical staining

**Fig. 1:**
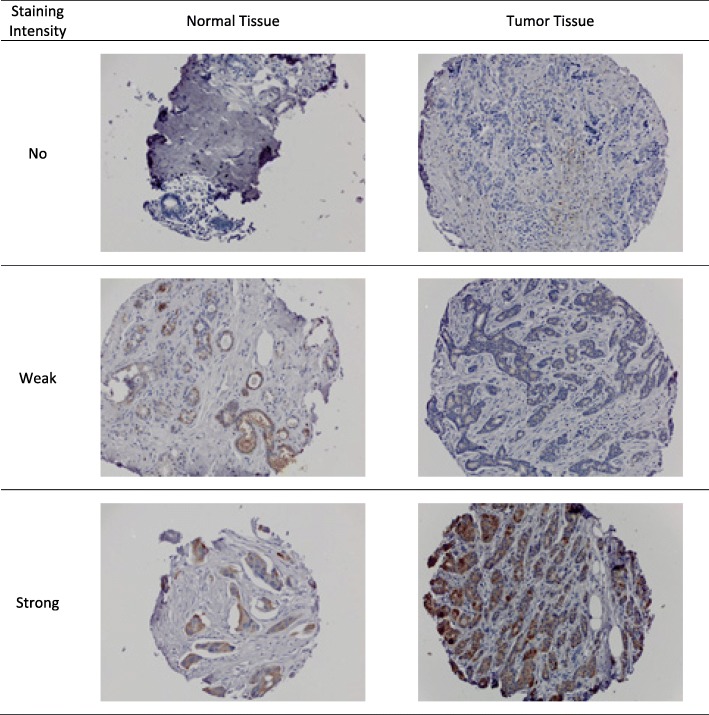
Example of TMA Staining for COX-2

Following preparation of slides from the TMAs, staining with antibodies against monoclonal COX-2 (dilution 1:100]; clone SP21; Biocare Medical), monoclonal vimentin (dilution 1:200; clone 3B4; Dako), polyclonal IGF-1 (dilution 1:1000; Abcam), polyclonal IGF-1R (dilution 1:150; beta, clone C-20, Santa Cruz Biotechnology), polyclonal IGFBP-2 (dilution 1:25; Cell Signaling Technologies), monoclonal IGFBP-3 (dilution 1:50; Calbiochem), monoclonal STAT3 (dilution 1:50; clone E121–221; Abcam), polyclonal TNF-α (dilution 1:50 Abcam), polyclonal TGF-β (1:250 dilution Abcam), and monoclonal CD45 (dilution 1:100; clone 2B11 + PD7/26; DAKO) was performed. A board certified pathologist assessed cores with ≥25% epithelial tissue for the intensity of the staining in the majority of tumor or normal breast epithelial cells; a second pathologist evaluated a sample of markers for quality control by re-reading one slide per marker. Discrepant readings were reconciled but not quantitatively summarized. Identical intensity categories were used across markers (Fig. 1): no (0), weak (1), and strong (2). The highest category was assigned as summary score for each participant. Depending on the marker and tissue type, a substantial number of women had missing values due to the loss of cores during TMA construction, staining process, or lack of epithelial tissue for evaluation. Problems with the antibodies precluded any valid readings for IGF-1. IGFBP-3 did not have sufficient variation in staining results (186 women positive and 9 none) and CD45 did not show staining in any core. Robust staining for vimentin was due to the ubiquity of vimentin in stromal tissue. For STAT3 as well as TGF-β, TNF-α, IGF-1R, and IGFBP-2 in normal tissue, more than 140 (50%) of women had no readable cores. To allow for sufficient variation in statistical models, only markers with at least 20% positive marker expression and at least 10% negative expression were analyzed (COX-2, TGF-β, TNF-α, vimentin, IGF-1R, IGFBP-2).

### Statistical analysis

The statistical analysis was conducted with the SAS statistical software 9.4 (SAS Institute Inc., Cary, NC). Chi-square (χ^2^) tests were performed to estimate associations between markers and covariates and to compare ethnic groups. Spearman correlation coefficients were computed to assess the relation between marker expression in normal and malignant tissue. Multiple linear regression was applied to evaluate the relation of marker expression with mean percent density. From the regression models, we calculated the means of percent density for the three categories of marker expression (no, weak, strong) with 95% confidence intervals (CI) after having controlled for all covariates, i.e., holding them constant at their mean values. Trend tests were performed using the indicator variables (0–2) for marker status as a continuous variable. The first set of models was adjusted for age and BMI only and the final models for all covariates as assessed at cohort entry (except menopausal status and HT use) known to be predictors of MD (Additional file 1: Table S1): age at mammogram, BMI (< 25, 25- < 30, 30+ kg/m^2^), ethnicity (Caucasian, Native Hawaiian, Japanese, Other), parity, smoking (never, past, current), HT use (never, estrogen only, estrogen/progesterone), and years of NSAID use [2, 21]. Analyses stratified by menopausal status were also conducted.

## Results

The 279 study participants were 36–78 years old (mean: 57.6 ± 8.7 years) and predominantly postmenopausal (*N* = 203/279; 73%) with 97 (35%) Caucasians, 36 (13%) Native Hawaiians, 121 (43%) Japanese, and 25 (9%) other ethnic background (Table 1). The overall mean MD was 39.0 ± 22.6% with similar values in Caucasians and Japanese (38.4 ± 24.3 and 39.7 ± 21.4%) and a lower mean in Native Hawaiians (32.5 ± 19.1%). MD was lower for post- (35.9 ± 21.8%) than pre-menopausal women (47.2 ± 22.9%). Ethnic groups showed differences in their proportions of overweight and obese participants (χ^2^ test: *p* = 0.005). The majority of breast cancers were diagnosed at a localized stage (169/279 = 61%), followed by in situ (61/279 = 22%) and regional (37/279 = 13%); only 5 out of 279 (4%) were metastatic cancers.

**Table 1:** Characteristics of the 279 study participants

Characteristic		All
Sample size	*N* (%)	279 (100)
Age at mammography	Years ± std	57.6 ± 8.7
Mammographic density	Percent ± std	39.0 ± 22.6
Body mass index (%)	< 25 kg/m^2^	167 (60)
	25- < 30 kg/m^2^	81 (29)
	30+ kg/m^2^	31 (11)
Menopausal status (%)	Premenopausal	76 (27)
	Postmenopausal	203 (73)
Number of children (%)	0–1	92 (33)
	2–3	134 (48)
	> 3	53 (19)
Hormone use (%)	Never	102 (37)
	Estrogen only	97 (35)
	Estrogen and progesterone	80 (29)
Tumor stage (%)	In situ	61 (22)
	Localized	169 (61)
	Regional	37 (13)
	Distant	12 (4)

The proportion of women with missing readings varied between 29 and 253 (10–94%) per marker (Table 2). Markers with > 50% missing were not statistically evaluated. Having no evaluable cores was not related to mammographic density. The mean percent density values for the 8 markers were within 2% points of the overall mean (39.0 ± 22.6%): COX-2 (39.3 ± 22.9% in normal and 38.8 ± 21.7% in malignant tissue); TGF-β (39.4 ± 23.1%); TNF-α (38.0 ± 23.3%); vimentin (39.5 ± 22.6% in normal and 39.5 ± 22.7% malignant tissue); IGF-1R (39.9 ± 22.9%); IGFBP-2 (39.0 ± 22.9%).

**Table 2:** Results of immunohistochemistry for 279 study participants

Marker	No staining		Weak staining		Strong staining		Missing	
	*N*	%	*N*	%	*N*	%	*N*	%
COX-2: normal	50	18%	45	16%	78	28%	106	38%
COX-2: tumor	42	15%	40	14%	75	27%	122	44%
TGF-β: normal ^a^	27	10%	42	15%	15	5%	195	70%
TGF-β: tumor	94	12%	68	34%	85	24%	32	30%
TNF-α: normal ^a^	28	10%	43	15%	7	3%	201	72%
TNF-α: tumor	54	19%	99	36%	41	15%	85	30%
Vimentin: normal	50	18%	145	52%	33	12%	51	18%
Vimentin: tumor	47	17%	82	30%	121	43%	29	10%
IGF-1R: normal ^a^	25	9%	3	1%	10	4%	241	86%
IGF-1R: tumor	69	25%	58	21%	73	26%	79	28%
IGFBP-2: normal ^a^	8	3%	2	1%	6	2%	263	94%
IGFBP-2: tumor	51	18%	67	24%	66	24%	95	34%

^a^ These markers were not analyzed in relation to mammographic density because the proportion missing was > 50%

**Fig. 2:**
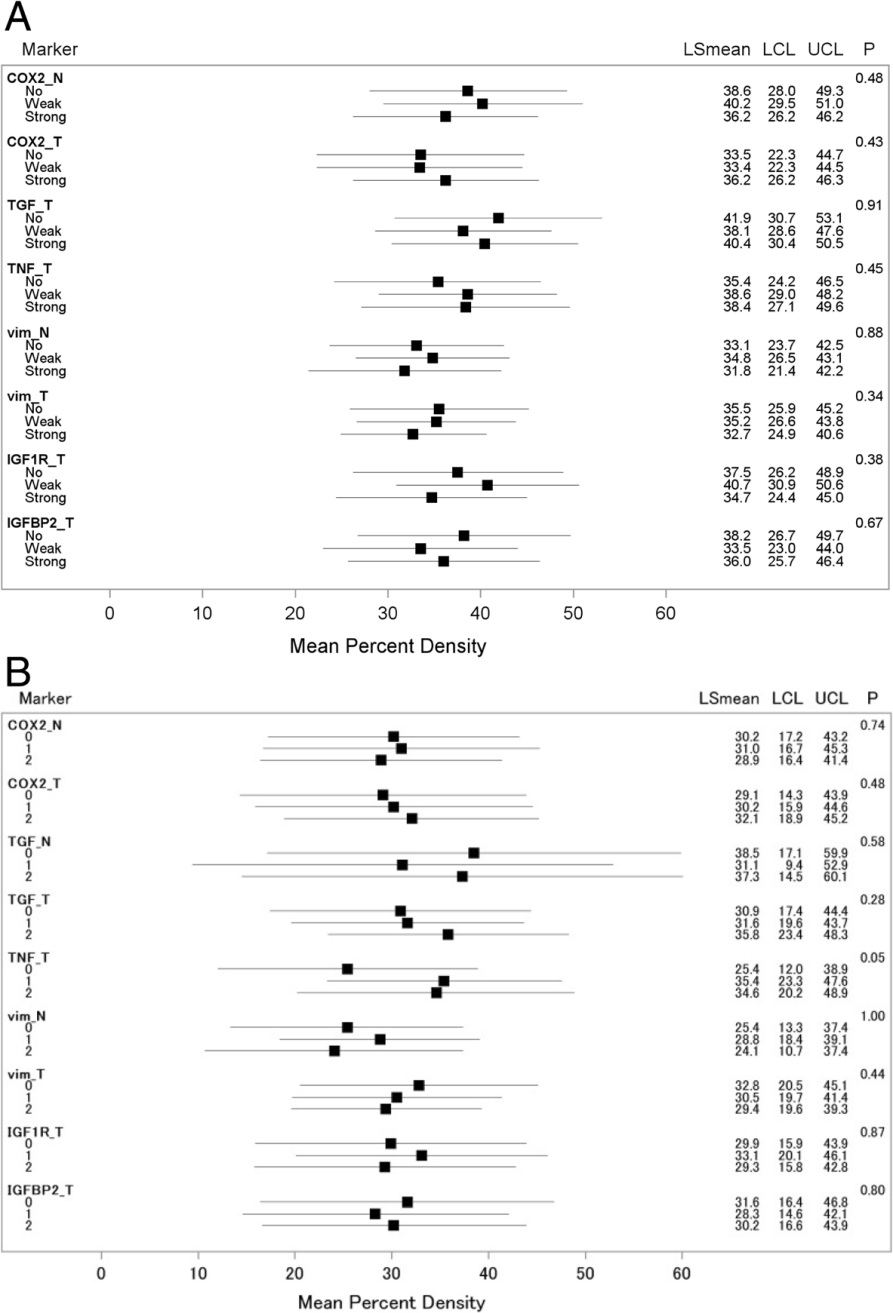
Mean mammographic density with 95% confidence intervals for each level of marker expression (no, weak, strong)^a^ (**a**) all women and (**b**) postmenopausal women. (^a^Means of percent density for the three categories of marker expression (no, weak, strong) were calculated by linear regression after having controlled for all covariates, i.e., holding them constant at their mean values. The *P*-values evaluate the significance of the trend tests. *Abbreviations*: N and T after the marker names indicate normal vs. tumor tissue, *vim* vimentin)

In simple models with adjustment for age and BMI only (Table 3), no significant associations with MD were observed. After adjustment for additional covariates, the values of the trend tests changed only slightly (Fig. 2) given that most associations between markers and covariates were not statistically significant (Additional file 1: Table S2).

**Table 3:** Association between immunohistochemical markers and percent density ^a^

Marker	No staining		Weak staining		Strong staining		*p*_trend_^b^
	Mean	95% CI	Mean	95% CI	Mean	95% CI	
COX2: normal	35.6	29.6, 41.5	35.8	29.3, 42.2	32.8	27.6, 38.0	0.43
COX2: tumor	30.5	23.9, 37.1	33.1	26.1, 40.1	35.2	30.2, 40.2	0.22
TGF-β: tumor	36.2	28.6, 43.7	32.4	27.8, 37.0	34.7	29.5, 40.0	0.95
TNF-α: tumor	30.5	24.4, 36.6	34.2	29.6, 38.7	32.9	26.3, 39.4	0.54
Vimentin: normal	32.9	27.1, 38.7	33.9	30.1, 37.7	31.6	24.5, 38.6	0.83
Vimentin: tumor	35.4	29.2, 41.6	35.0	30.2, 39.7	32.9	28.9, 36.8	0.40
IGF-1R: tumor	32.6	27.1, 38.0	37.7	32.1, 43.2	30.4	25.4, 35.4	0.51
IGFBP-2: tumor	36.7	30.5, 42.9	31.7	26.1, 37.4	33.8	28.4, 39.2	0.52

^a^Adjusted means were obtained from linear regression adjusted for age and BMI

^b^p_trend_ obtained from linear regression using staining (0–2) as continuous variable

COX-2 staining (0–2) was similar in normal and malignant tissue (Table 2) with a correlation of 0.24 (95% CI 0.04–0.43) and demonstrated no clear association with MD. The respective mean MD values for none, weak, and strong expression (Fig. 2) were 38.6% (95% CI 28.0–49.3%), 40.2% (95% CI 29.5–51.0%), and 36.2% (95% CI 26.2–46.2%) in the fully adjusted model. The differences across staining categories for COX-2 and TGF-β in tumor tissue were equally small and non-significant. Although TNF-α in tumor tissue was not associated with MD showing mean values of 35.4% (95% CI 24.2–46.5%), 38.6% (95% CI 29.0–48.2%), and 38.4% (95% CI 27.1–49.6%), a *posthoc* analysis restricted to postmenopausal women indicated greater percent density across categories of TNF-α expression with values of 25.4% (95% CI 12.0–38.9%), 35.4% (95% CI 23.3–47.6%), and 34.6% (95% CI 20.2–48.9%).

Tumor tissue (Table 2) had a higher proportion of women with strong vimentin expression than normal tissue 121/279 (43%) vs. 33/279 (12%); the correlation between normal and malignant tissue was 0.11 (95% CI -0.02-0.24). There was no substantial associations between vimentin and MD in normal as demonstrated by the minimal differences across staining categories (Fig. 2). Stratification by menopausal status did not modify these associations substantially.

Growth factor expression (Fig. 2) also did not demonstrate any association with MD. The small differences by expression of IGF-1R in tumor tissue were not significant (37.5% [95% CI 26.2–48.9%], 40.7% [95% CI 30.9–50.6%], and 34.7% [95% CI 24.4–45.0%]). Similarly, the IGFBP-2 expression in tumor tissue varied without a clear trend (38.2% [95% CI 26.7–49.7%], 33.5% [95% CI 23.0–44.0%], and 36.0%; [95% CI 25.7–46.6%]). There was also no association after stratification by menopausal status.

## Discussion

The current study detected little support for an association between the expressions of selected histopathologic markers in breast tissue with MD. Only for TNF-α, a significant positive association between expression in tumor tissue and MD among postmenopausal women was detected, but this may be a false positive result due to multiple testing. Although the differences were not statistically significant, mean MD was also higher with stronger expression of COX-2 and TGF-β in tumor tissue. These findings offer weak support for the role of inflammation as a possible pathway for high mammographic density. No associations for IGFBP-2, IGF-1R, and vimentin were observed.

In comparison to the literature, a positive association between TNF-α expression and MD in premenopausal women but not postmenopausal women disagrees with the current finding [22], but an association of TNF genetic variants with MD in postmenopausal women who never used HT is in support [23]. As to COX-2, the current results align with previous studies [22, 24, 25], which did not find stronger expression in women with high MD. In contrast, a relation was detected in epithelial cells from normal breast tissue of high-risk women who underwent a prophylactic mastectomy [25]. However, this study examined high and low MD regions within individual breasts and did not investigate differences across women. Similarly, lower TGF-β mRNA levels found in normal breast tissue in women with dense breasts [24] contradict our null results, but the correlation between mRNA and protein levels of TGF-β may be different. Our analysis of the IGF-axis markers IGFBP-2 and IGF-1R did not suggest an association between their expression and MD. To our knowledge, the relation between IGFBP-2 and MD has not been previously investigated, but a report from women with breast cancer in Korea [26] found that IGF-1R overexpression in normal breast tissue was related to denser breasts. In comparison to our study, the population was younger (median: 47 years) and had a higher proportion of premenopausal women (59%). The expression of IGF-1R in tumor tissue may differ from normal breast tissue or pathways involving IGF-1R may differ by menopausal status. As vimentin is ubiquitous in stromal tissue, our hypothesis of higher MD women with stronger expression was not confirmed. No previous results have been published.

Inflammation as a possible mechanism for higher MD is supported by several observations. TNF-α, a cytokine produced primarily by macrophages and monocytes has been implicated in the progression of breast cancer and promotion of tumor growth by increasing estrogen production [27]. Given that our significant finding was limited to postmenopausal women when breast adipose tissue provides most of the estrogens [28], TNF-α may upregulate aromatase and increase the activity of estrone sulfatase through post-translational modifications [27] among other possible pathways [29]. One hypothesized mechanism for the tumorigenic effects of COX-2 is also upregulation of aromatase P450 and increasing the biosynthesis of estrogen [30]. COX-2 expression in stromal, but not epithelial, tissue was associated with overall survival in breast cancer patients, suggesting a distinction between stromal and epithelial in COX-2 action [31]. Thus, the association between COX-2 and MD may be restricted to stromal tissue. TGF-β is involved in regulating cell proliferation and apoptosis and plays a role in inflammation, e.g., in the maturation of B lymphocytes [32], but it can act pro-oncogenic or as a tumor suppressor [33].

Inconsistent findings on the relationship between IGFBP-2 and breast cancer prognosis have been reported previously. A large study of breast cancer patients did not find that IGFBP-2 expression was associated with adverse survival except among hormone receptor-negative patients [34]. However, IGFBP-2 expression in breast tissue was associated with better survival among more than 885 women with primary breast cancer with possible effect modification by BMI [10]. Ethnicity interacted with IGFBP-2 expression in a study from Hawaii; Native Hawaiians with IGFBP-2 expression experienced a greater risk for mortality, but this could be due to the higher rates of obesity in this ethnic group [35]. These different findings may reflect the complexity of the effects of the IGFBPs on cell growth, which can be either stimulatory or inhibitory depending on interactions with signaling systems [36]. It appears that IGF-1R expression and prognosis depends on the hormone status of the breast cancer [9]. Ethnicity, specifically Native Hawaiian, also appears to increase the risk of mortality for women expressing IGF-1R in the breast [35]. The activation of IGF-1R by binding with IGF-1 or IGF-2 can result in a complex cascade of signaling pathways involving cell growth, survival, and motility [37].

While classically known as a marker of the epithelial-mesenchymal transition [38] and indicating the presence of stromal tissue, an important component of MD, vimentin may also indicate local inflammation as shown by the presence of CD45 immune cells with vimentin in the epithelial layer [15]. Although vimentin expression was not associated with mammographic density, which disagrees with the one previous report [15], we observed stronger vimentin expression in tumor tissue than in normal (43 vs. 12% of women). This is in accordance with several laboratory investigations that have found overexpression correlated with increased cell motility and invasiveness in breast cancer cells. As abnormal vimentin expression during the epithelial–mesenchymal transition has been regarded as an important element for epithelial plasticity and tumor metastasis, variation in the epithelial contribution to MD may obscure the relation between vimentin and MD.

Strengths of the current study include the availability of many covariates, the high quality of MD assessment, and the ethnic diversity of the population. Immunohistochemical staining of breast tissue allowed for evaluation of local biomarker expression, which is likely more relevant to biological processes than circulating levels. Weaknesses were introduced by the limited statistical power based on the small sample size. The use of small tissue cores (0.6 mm) on the TMAs led to the loss of specimens during staining and a lack of epithelial cells for evaluation in many cores, in particular for normal tissue. Therefore, our analysis was primarily based on tumor tissue. Only vimentin and COX-2 had sufficient staining in normal tissue while the expression of TNF-α, TGF-β, IGFBP-2, IGF-1R was analyzed in cores of tumor tissue only, a potential problem as the histopathological profile of the tumor does not necessarily reflect the conditions of the normal breast tissue representing MD in healthy breasts. The tissue considered normal in our study originated from surgery specimens and may be influenced by paracrine signals from the nearby tumor tissue; thus it is probably different from “normal” tissue in women who never develop breast cancer. Despite the large number of missing readings for several markers, we did not detect an association between missing status and MD. Multiple testing is another serious issue in our analysis and may be responsible for the single significant finding among close to 20 models. In conclusion, the current analysis offers little support for the hypothesis that several immunohistochemical markers representing inflammatory and growth factor pathways are predictors of breast density.

## Additional file

Additional file 1: **Table S1.** Residual Association of Covariates with Percent Density^a^. **Table S2.** Association of Markers with Covariates^a^. (DOCX 13 kb)
