# Supplementary material for: Mammographic density and breast tissue expression of inflammatory markers, growth factors, and vimentin
Source: BMC Cancer. 2018 Nov 29;18:1191. doi: 10.1186/s12885-018-5088-9 (PMC6267911; doi:10.1186/s12885-018-5088-9)
Supplement: Supplementary file 1 — Table S1. Residual Association of Covariates with Percent Densitya. Table S2. Association of Markers with Covariatesa. (DOCX 13 kb) [file 12885_2018_5088_MOESM1_ESM.docx]

Table S1. Residual Association of Covariates with Percent Density^a^

| Marker | Type III SS | Mean square | F-value | p |
| --- | --- | --- | --- | --- |
| Ethnicity | 2813 | 938 | 2.31 | 0.08 |
| Hormone use | 5072 | 2536 | 6.39 | 0.002 |
| Smoking | 726 | 363 | 0.88 | 0.42 |
| Parity | 1258 | 1258 | 3.07 | 0.08 |
| NSAID use | 3235 | 809 | 1.98 | 0.10 |

^a^Obtained by linear regression models adjusted for age and BMI

Table S2. Association of Markers with Covariates^a^

| Marker | Age | Ethnicity | BMI | Hormone use | Smoking | Parity | NSAID use |
| --- | --- | --- | --- | --- | --- | --- | --- |
| COX2: normal | 0.17 | 0.88 | 0.12 | 0.20 | 0.54 | 0.95 | 0.79 |
| COX2: tumor | 0.38 | 0.21 | 0.39 | 0.09 | 0.29 | 0.50 | 0.94 |
|  |  |  |  |  |  |  |  |
| TGF-β: tumor | 0.55 | 0.28 | 0.84 | 0.96 | 0.07 | 0.49 | 0.35 |
|  |  |  |  |  |  |  |  |
| TNF-α: tumor | 0.19 | 0.92 | 0.55 | 0.45 | 0.04 | 0.34 | 0.58 |
|  |  |  |  |  |  |  |  |
| Vimentin: normal | 0.05 | 0.80 | 0.37 | 0.32 | 0.80 | 0.05 | 0.85 |
| Vimentin: tumor | 0.18 | 0.87 | 0.33 | 0.31 | 0.18 | 0.29 | 0.87 |
|  |  |  |  |  |  |  |  |
| IGF-1R: tumor | 0.14 | 0.05 | 0.27 | 0.61 | 0.85 | 0.38 | 0.69 |
|  |  |  |  |  |  |  |  |
| IGFBP-2: tumor | 0.31 | 0.67 | 0.43 | 0.58 | 0.51 | 0.53 | 0.31 |

^a^P-values of chi-square tests are shown
